# Case of Strangulated Femoral Hernia Successfully Treated by Opium

**Published:** 1847-10

**Authors:** Charles Mayo


*Case of Strangulated Femoral Hernia successfully treated by Opium.* By Charles Mayo, Esq., F. R. C. S., &c. Mrs. D., aged about 67, became subject to femoral hernia on the right side about four years ago, at which time it was strangulated, and after some trouble I succeeded in reducing it by the taxis. Since that time she had worn a truss, and was careful to keep it reduced. The truss had now become broken and nearly useless. After some unusual exertion on the morning of the 24th of April, she felt a large portion of bowel suddenly to protrude, she became sick, took opening pills, and laid up. On the 25th she sent to me; I found the swelling as large as an egg, painful, and tender, from her having used much exertion in endeavouring to reduce it, or push it back, as she said. She was constantly sick. I gave her a cathartic enema, and used the taxis without effect. I then left her six pills with a grain of opium in each, desiring her to take one every hour till I saw her again, beginning at 4 P. M. At nine o'clock I found that she had taken four of the pills. that the vomiting had ceased after the first, and that she was quite easy; cold cloths were kept applied to the swelling, which remained immoveable. As she was so easy, I advised the two remaining pills to be taken at intervals of four hours, another glyster to be thrown up in the course of the night, and a cathartic draught to be given at six in the morning.

April 26th. I received a message this morning that Mrs. D. was completely relieved, and on my calling about twelve, I found that the fifth pill was taken at midnight, and the sixth at four in the morning; after this she felt completely relaxed all over, her bowels rumbled about, and the swelling seemed to be enlarged and distended with wind, but soon after on feeling it with her hand, it had become softer, and presently went entirely up under very slight pressure. She took the draught at six, it had operated satisfactorily, and she was delighted to sleep all the day after. I was not less pleased to have the necessity for an operation to be superseded, which I had the day before considered as nearly inevitable. Dr. Butler Lane has so well set forth the *modus operandi* of this remedy that I have nothing more to add, than that if you consider this communication to be of any use as an encouragement to others to make such trials, it is quite at your service.—*Prov. Med. Jour.*

